# High-performance method for identification of super enhancers from ChIP-Seq data with configurable cloud virtual machines

**DOI:** 10.1016/j.mex.2020.101165

**Published:** 2020-11-28

**Authors:** Natalia N. Orlova, Olga V. Bogatova, Alexey V. Orlov

**Affiliations:** a Moscow Institute of Physics and Technology (State University), Dolgoprudny, Moscow Region, Russia; b Prokhorov General Physics Institute of the Russian Academy of Sciences, Moscow, Russia

**Keywords:** Epigenomics, Chromatin immunoprecipitation followed by sequencing, Next generation sequencing, Stitched enhancers, H3K27ac

## Abstract

A universal method for rapid identifying super-enhancers which are large domains of multiple closely-spaced enhancers is proposed. The method applies configurable cloud virtual machines (cVMs) and the rank-ordering of super-enhancers (ROSE) algorithm. To identify super-enhancers a сVM-based analysis of the ChIP-seq binding patterns of the active enhancer-associated mark is employed. The use of the proposed method is described step-by-step: configuration of cVM; ChIP-seq data alignment; peak calling; ROSE algorithm; interpretation of the results on a client machine. The method was validated for the search of super-enhancers using the H3K27ac mark in the sample datasets of a cell line (human MCF-7), mouse tissue (heart), and human tissue (adrenal gland). The total analysis cycle time of raw ChIP-seq data ranges from 15 to 48 min, depending on the number of initial short reads. Depending on the data processing step and availability of multi-threading, a cVM can be scaled up to a multi-CPU configuration with large amount of RAM. An important feature of the method is that it can run on a client machine that has low-performance with virtually any OS. The proposed method allows for simultaneous and independent processing of different sample datasets on multiple clones of a single cVM.

• Cloud VMs were used for rapid processing of ChIP-seq data to identify super-enhancers.
• The method can use a low-performance computer with virtually any OS on it.
• It can be scaled up for parallel processing of individual sample datasets on their own VMs for rapid high-throughput processing.

## Graphical abstract

**Figure:**
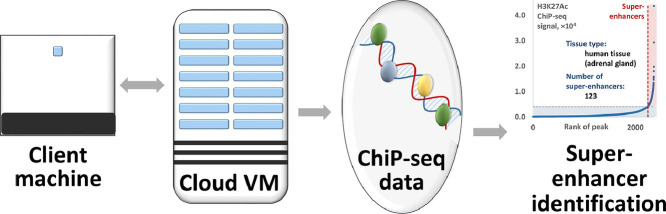

Specifications

**Table:** 

Subject Area:	Bioinformatics
More specific subject area:	Methods for chromatin immunoprecipitation followed by sequencing (ChIP-seq) analysis
Method name:	Identification of super enhancers from ChIP-seq data with configurable cloud virtual machines
Name and reference of original method:	Rank Ordering Of Super-Enhancers (ROSE) algorithm [1,2]
Resource availability:	https://github.com/ChIP-seq/cloudROSE

## Method details

### Introduction

A novel modern epigenomics approach which was introduced in 2013 but has already proven to be highly efficient, is the identification of super-enhancers – large domains of multiple closely-spaced enhancers [1], [2], [3]. These sites play an important role in the transcriptional regulation in many diseases [4], [5], [6], [7]. Super-enhancers have been demonstrated to be a promising target in the studies of cancer (acute myeloid leukemia [8], neuroblastoma [9], etc.) and a wide range of other disorders, such as type 1 diabetes [3], Alzheimer's disease [10] and multiple sclerosis [11]. However, the existing methods for identification of super-enhancers are time-consuming and resource-intensive. In this work, we propose a fast method for super-enhancer identification using a low-performance computer based on configurable cloud virtual machines (cVMs) and the Rank-Ordering of Super-Enhancers (ROSE) algorithm.

### Creation of a virtual machine

Currently, there is a number of various cloud-based virtual machine services, including commercial projects such as Amazon Web Services (AWS), Microsoft Azure and Google Cloud Platform (GCP) [12], [13], [14]. In this work, we used cVMs created with Yandex Compute Cloud based on CentOS 8 with 200 GB HDD. Detailed guidelines to set up a cVM are usually released by the service that provides the virtual machine. In particular, for the cVMs used in this work these instructions are described in Ref. [15].

Importantly, one can manage a cVM using a low-performance client machine running virtually any OS. To demonstrate this feature, we performed all operations on a laptop with a single 1.9 GHz core and 2 GB RAM. The cVM configuration (number of CPUs and the amount of RAM) was set on-demand depending on the stage of the method and is described below. When creating a cVM, we chose a cost-efficient configuration with 2 CPUs and 2 GB RAM. To speed up the processing of multiple datasets, a pre-configured cVM was cloned to operate multiple instances of one cVM simultaneously and independently. This feature dramatically reduces the total processing time for large numbers of sample datasets. Notably, cVMs could be created using almost any services that support the SSH protocol to implement the proposed method.

### SSH key pair generation

Depending on the client machine OS, the procedure for generating an SSH key pair is described below.

#### Linux/MacOS/Windows 10

To generate public/private key pairs use *ssh-keygen* command in a terminal/command prompt:

$ ssh-keygen -t rsa -b 2048

Enter the names of the files where the keys are to be saved and enter the private key password. The default file name is *id_rsa*, and the keys are created in the *~/.ssh directory* on Linux/MacOS and in the *C:\Users\<user_name>\.ssh\* on Windows 10. The public part of the key is saved in a file *〈key_name〉.pub*. Paste this key into the SSH key field when creating a new cVM via the console.

#### Windows 7/8

SSH keys for Windows 7/8 are generated using the PuTTY SSH client [16]. Select key type as RSA and 2048 as the key size. Press *Generate* and keep moving your mouse over the blank area until the key is complete. Enter password in *Key passphrase* and click *Save private key*. Copy the contents of your public key from the text box to a text file and enter it when creating a cVM via the console.

### Connection to a virtual machine

Start the cVM instance through the console and use for connection the SSH client on Linux/macOS/Windows 10, or PuTTY on Windows 7/8. In order to connect to a cVM, provide the public address of the cVM. Depending on the client machine OS, the procedure for connecting to a cVM is as follows:

#### Linux/MacOS/Windows 10

Run the following command in a terminal/command prompt:

$ ssh -i〈path/to/id_rsa〉 <user_name>@<cVM IP address or name>

If you are connecting to the cVM for the first time, you might get the ‘unknown host’ warning.

#### Windows 7/8

Use PuTTY SSH client to connect to a cVM on Windows 7/8. Start the Pageant application and select *Add key* in the context menu. Select the generated by PuTTY *.ppk* private key and enter the password. Then, launch PuTTY and enter the public IP address of the cVM in *Host Name* (or *IP address*). Enter port 22 and SSH connection type. Open *Connection - SSH - Auth* on the left-hand side of the tree. Check box *Allow agent forwarding*. In the *Private key file for authentication* field, select the private key file and click *Open*. If you are connecting to the cVM for the first time, you might get the ‘unknown host’ warning.

### Installation of updates and required packages

Before data processing with a newly created cVM, install the updates and required packages:

$ sudo yum update
$ sudo yum install wget gcc unzip bzip2 tar make git

Note that *yum* commands is mainly used in CentOS, for Debian/Ubuntu systems use *apt-get* instead.

### Download and quality assessment of ChIP-Seq raw data

In this work, we used the ChIP-Seq data from the Encyclopedia of DNA Elements (ENCODE) public database. The data was retrieved onto the cVM using the command wget from the ENCODE Project Portal: https://www.encodeproject.org. All the data that we used here are listed in the Method Validation section. The following test/control sample datasets were used: cell line (human MCF-7), ENCFF000VHD/ENCFF000VHL; mouse tissue (heart), ENCFF483VRP/ENCFF183PVA; and human tissue (adrenal gland), ENCFF697BQR/ENCFF225QVL.

The ChIP-seq raw data is analyzed on a client machine using FastQC quality control tool [17].

### Alignment with bowtie2

The first step in processing ChIP-seq raw data is to align them with a reference genome. For this purpose, we used *bowtie2* tool for aligning sequencing reads [18] which we installed using *bioconda* package [19] preinstalled on the cVM:

$ wgethttps://repo.anaconda.com/miniconda/Miniconda3-latest-Linux-x86_64.sh
$ sh Miniconda3-latest-Linux-x86_64.sh

Set the path to *conda* with the command:

$ export PATH=$PATH:/path/to/conda/bin

Then, we installed *bowtie2*:

$ conda install -c bioconda bowtie2

To use bowtie2, one first needs the index of the reference genome for the corresponding ChIP-seq data. To index a genome, one can use a built-in command *bowtie2-build* [20], or can download pre-indexed genomes at http://bowtie-bio.sourceforge.net/bowtie2. We worked with Mus musculus (house mouse) genome (mm10) and Homo sapiens (human) genome GRCh38, the indices of which were obtained with the commands:

$ wgethttps://genome-idx.s3.amazonaws.com/bt/mm10.zip
$ wgethttps://genome-idx.s3.amazonaws.com/bt/GRCh38_noalt_as.zip

Genome alignment is relatively time-consuming and resource-intensive. Therefore, before starting the analysis, we reconfigured the сVM in order to speed up the alignment via multi-threading. In particular, we increased the number of CPUs to 64 and the amount of RAM to 256 GB. Then, we started the alignment with the following command:

$ bowtie2 -p 64 -q –local -x〈bt2-index〉 −1〈path to File 1〉 −2〈path to File 2〉 -S〈path to output file〉

Here, *〈bt2-index〉* is the basename of the index for the reference genome; *〈path to File 1〉* is the path to the paired-end FASTQ File 1; *〈path to File 2〉* is the path to the paired-end FASTQ File 2. If the data were unpaired and contained in a single file, then instead of *‘−1 〈path to File 1〉 −2 <path to File 2>*’, use “–*U <path to File>”.* As a result, a SAM file is received that was subsequently converted and sorted using *samtools* [21].

### Conversion and sorting using samtools

*Samtools* were installed on cVM using *bioconda*:

$ conda install -c bioconda samtools

Note that *samtools* depends on library *libncurses.so.5*. Therefore, if *ncurses6.2* is installed than create a symbolic link for *libncurses.so.5* to *libncurses.so.6*:

$ ln -s ~/miniconda3/lib/libncurses.so.6.2 ~/miniconda3/lib/libncurses.so.5

The SAM file obtained by *bowtie2*, we converted to a BAM file:

$ samtools view -S -b - @ 64〈path to SAM File〉 〈path to BAM File〉

Then, we sorted the BAM file:

$ samtools sort -o - @ 64〈path to sorted BAM File〉 〈path to BAM File〉

Based on the sorted BAM file, one can generate an index file for fast random access using the command below:

$ samtools index〈path to sorted BAM File〉

### Peak calling with Macs2

Peak calling was performed using the MACS algorithm (Model-Based Analysis of ChIP-Seq) [22], [23], [24] in MACS2 2.2.7.1 [25]. We installed MACS2 as follows:

$ wget https://files.pythonhosted.org/packages/e2/61/85d30ecdd34525113e28cb0c5a9f393f93578165f8d848be5925c0208dfb/MACS2–2.2.7.1.tar.gz
$ tar zxvf MACS2–2.2.7.1.tar.gz
$ cd MACS2–2.2.7.1
$ python setup.py install

To run MACS2, one need to process two ChIP-seq data sets as described above and get two sorted BAM files — for a test sample and for a control sample. Then, run MACS2 as follows:

$ macs2 callpeak -p 1e-9 –keep-dup=auto -t〈path to the BAM-sorted test sample file〉 -c〈path to the BAM-sorted control sample file〉 -n〈output path〉

### Identification of super-enhancers with ROSE

To find and identify super-enhancers, we used the Rank Ordering of Super-Enhancers (ROSE) algorithm [1,2]. It requires Python 2.7 which was pre-installed as follows:

$ wgethttps://www.python.org/ftp/python/2.7.18/Python-2.7.18.tgz
$ tar xzf Python-2.7.18.tgz
$ cd Python-2.7.18
$ ./configure –enable-optimizations
$ sudo make altinstall

ROSE-package was cloned from the Barsky lab:

$ git clonehttps://github.com/Barski-lab/ROSE.git

To start ROSE, use the command:

python2.7 〈ROSE path〉 /ROSE_main.py -g 〈genome build: one of hg18, hg19, mm8, mm9, or mm10〉 -i 〈peaks.bed path created by MACS2〉 -r 〈path to BAM-sorted test sample file〉 -c 〈path to the BAM-sorted control sample file〉 -o 〈output path〉 -s 12,500 -t 2000

### Processing of results

After processing the data in the cVM, we transferred the files with the results to the client machine using FileZilla and processed them with standard tools such as IGV Browser, Excel, etc. All output files obtained as a result of the ROSE algorithm are available on the resource: https://github.com/ChIP-seq/cloudROSE.

### Method validation

To demonstrate the capabilities of the method, we performed a search for super-enhancers in three different sample types – a cell line (human MCF-7), a mouse tissue (heart), and a human tissue (adrenal gland). The acetylation at the 27th lysine residue of the histone H3 protein (H3K27Ac) was used as an enhancer mark. Table 1 provides information on the ChIP-seq data from the ENCODE Project that we used.

**Table 1:** Information about ChIP-seq data used.

Dataset ID	Sample Type	ChIP-seq Mark	Platform	Reference Genome
ENCFF000VHD	Cell line (human MCF-7)	H3K27Ac	Illumina Genome Analyzer	GRCh38
ENCFF000VHL	Cell line (human MCF-7)	Control (input library)	Illumina Genome Analyzer	GRCh38
ENCFF483VRP	Mouse tissue (heart)	H3K27Ac	Illumina HiSeq 4000	Mm10
ENCFF183PVA	Mouse tissue (heart)	Control (input library)	Illumina HiSeq 4000	Mm10
ENCFF697BQR	Human tissue (adrenal gland)	H3K27Ac	Illumina HiSeq 2500	GRCh38
ENCFF225QVL	Human tissue (adrenal gland)	Control (input library)	Illumina HiSeq 2500	GRCh38

Table 2 shows the cVM configurations at each step of data processing using the developed method. cVM configuration was chosen in order to optimize the processing time, taking into account the possibility of multi-threading at a particular stage. We increased the number of CPUs and the amount of RAM as long as it still resulted in shorter processing times.

**Table 2:** Cloud VM configurations at each step of data processing using the developed method.

	Alignment (bowtie2)	Conversion (samtools)	Sorting (samtools)	Peak Calling (MACS2)	Identified Super-Enhancers (ROSE)
**CPU**	64	8	8	8	4
**RAM**	256	16	16	16	8

**Fig. 1:**
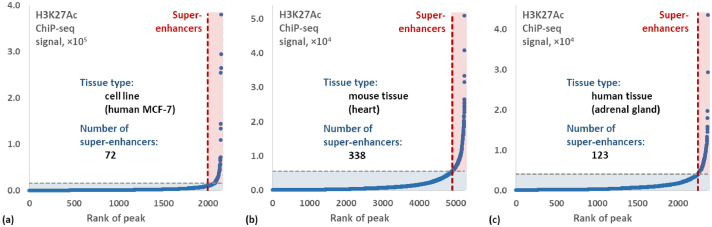
Dependence of normalized signal on the peak rank: the ChIP-Seq data ROSE processing results using the H3K27Ac enhancer mark in (a) MCF-7 human cell line; (b) mouse heart; and (c) human adrenal gland.

Table 3 shows method operating time at each of the processing steps and the number of potential super enhancer. Fig. 1 shows the results of ROSE for each of three sample types – cell line (human MCF-7), mouse tissue (heart), and human tissue (adrenal gland). The full processing time of raw ChIP-seq raw data ranged from 14 min 37 s to 48 min 03 s for a test/control pair of sample datasets depending on the number of the initial short reads. As one can see from the table, the processing time at each step was almost linear to the number of the short reads in the ChIP-seq raw data.

**Table 3:** The processing time of the method at each data processing step and the number of identified super-enhancers.

Sample Type	Test/ Control	Number of Reads	Processing time						Potential super-enhancers
			Alignment (bowtie2)	Conversion (samtools)	Sorting (samtools)	Peak calling (MACS2)	Identification of super-enhancers (ROSE)	Total	
Cell line (human MCF-7)	Test	31,418,572	02 m 42s	00 m 43s	00 m 37s	03 m 42s	05 m 30s	14 m 37s	72
	**Control**	18,714,964	00 m 44s	00 m 23s	00 m 16s				
Mouse tissue (heart)	Test	36,752,873	03 m 48s	00 m 57s	00 m 50s	11 m 09s	20 m 32s	48 m 03s	338
	**Control**	85,608,717	06 m 53s	02 m 17s	01 m 37s				
Human tissue (adrenal gland)	Test	33,443,289	04 m 12s	01 m 06s	00 m 56s	06 m 43s	10 m 27s	30 m 23s	123
	**Control**	41,510,868	04 m 45s	01 m 20s	00 m 54s

It should be noted that the stage of alignment reads is traditionally one of the most time-consuming steps in many genomics pipelines. With the developed method, it took a relatively short period of time – from 44 s for the sample dataset of 18.7 million reads to 6 min 53 s for the sample dataset of 85.6 million reads. This is primarily due to the efficient multi-threading algorithms underlying the *bowtie2* tools which were implemented on a cVM with 64 CPUs.

At the same time, as can be seen from Table 3, the ROSE algorithm turned out to be the slowest, most likely due to the fact that it is least efficient with multi-threading. Growing each cVM performance by increasing the number of CPUs or RAM amount did not significantly accelerate the ROSE. Nevertheless, the proposed method allows for simultaneous and independent processing of different sample datasets on different cVMs. To achieve this, the configured cVM is cloned in the required number of instances, and an individual cVM clone processes one dataset of test/control pair.

In our case, we ran ROSE algorithm simultaneously for all sample datasets on several low-performance cVMs instances. Here, the total processing time of all samples was actually equal to the processing time of one (the slowest) sample dataset. Parallel and independent processing of individual sample datasets on their own cVMs is one of the significant advantages of the proposed method that can be used for rapid high-throughput processing. Besides, due to the universal concept of a cloud virtual machine, the proposed method can be scaled up to address other resource-intensive biocomputing applications for analysis of genome-wide sequencing data. The method can be also used for the optimization of the ChIP-seq assay and for developing multiplex biosensing systems that provide highly sensitive detection of the agents essential in medical diagnostics [[26], [27]], veterinary medicine, food quality control [[28], [29]], environmental monitoring [30] and other applications.

## Declaration of Competing Interest

The authors declare that they have no known competing financial interests or personal relationships that could have appeared to influence the work reported in this paper.
